# The sequence and thresholds of leaf hydraulic traits underlying grapevine varietal differences in drought tolerance

**DOI:** 10.1093/jxb/eraa186

**Published:** 2020-04-11

**Authors:** Silvina Dayer, José Carlos Herrera, Zhanwu Dai, Régis Burlett, Laurent J Lamarque, Sylvain Delzon, Giovanni Bortolami, Hervé Cochard, Gregory A Gambetta

**Affiliations:** 1 EGFV, Bordeaux-Sciences Agro, INRA, Université de Bordeaux, ISVV, Villenave d’Ornon, France; 2 Institute of Viticulture and Pomology, University of Natural Resources and Life Sciences (BOKU), Tulln, Austria; 3 Beijing Key Laboratory of Grape Science and Enology and Key Laboratory of Plant Resources, Institute of Botany, the Chinese Academy of Sciences, Beijing, China; 4 Biodiversité Gènes et Communautés, Institut National de la Recherche Agronomique (INRA), Université Bordeaux, Cestas, France; 5 SAVE, INRA, BSA, ISVV, Villenave d’Ornon, France; 6 Université Clermont-Auvergne, INRA, PIAF, Clermont-Ferrand, France; 7 University of Cambridge, UK

**Keywords:** Drought, embolism, grapevine, stomata, transpiration, turgor loss point

## Abstract

Adapting agriculture to climate change is driving the need for the selection and breeding of drought-tolerant crops. The aim of this study was to identify key drought tolerance traits and determine the sequence of their water potential thresholds across three grapevine cultivars with contrasting water use behaviors, Grenache, Syrah, and Semillon. We quantified differences in water use between cultivars and combined this with the determination of other leaf-level traits (e.g. leaf turgor loss point, π _TLP_), leaf vulnerability to embolism (P_50_), and the hydraulic safety margin (HSM P_50_). Semillon exhibited the highest maximum transpiration (*E*_max_), and lowest sensitivity of canopy stomatal conductance (*G*_c_) to vapor pressure deficit (VPD), followed by Syrah and Grenache. Increasing *E*_max_ was correlated with more negative water potential at which stomata close (P_*gs*90_), π _TLP_, and P_50_, suggesting that increasing water use is associated with hydraulic traits allowing gas exchange under more negative water potentials. Nevertheless, all the cultivars closed their stomata prior to leaf embolism formation. Modeling simulations demonstrated that despite a narrower HSM, Grenache takes longer to reach thresholds of hydraulic failure due to its conservative water use. This study demonstrates that the relationships between leaf hydraulic traits are complex and interactive, stressing the importance of integrating multiple traits in characterizing drought tolerance.

## Teaser

The relationships between traits including stomatal regulation, leaf embolism thresholds, and survival time under drought are complex and interactive, stressing the importance of integrating multiple traits in characterizing drought tolerance.

## Introduction

In the context of climate change, the identification of key traits responsible for drought-tolerant species/genotypes has become a major challenge in plant research. There has been a huge emphasis placed on genetics and genomics, but both of these approaches rely upon accurate identification and phenotyping of the underlying physiological traits (Cattivelli *et al.*, 2008). Most studies aimed at the identification of vulnerable/tolerant genotypes are based on only one or a few traits (Tardieu and Simmoneau, 1998; Schultz, 2003; Pou *et al.*, 2012; Hopper *et al.*, 2014) and are often constrained to moderate drought stress (i.e. a narrow range of water potentials; Gerzon *et al.*, 2015; Coupel-Ledru *et al.*, 2017). We know that the traits contributing to drought tolerance are complex, involving morphological, physiological, and hydraulic mechanisms (Hochberg *et al.*, 2017*a*). New state-of-the-art phenotyping approaches, including mini-lysimeter platforms (Coupel-Ledru *et al.*, 2016, 2017; Charrier *et al.*, 2018), hold promise to more precisely phenotype these traits. However, different phenotyping approaches need to be integrated to assess multiple traits across a full range of drought stress (i.e. from moderate to severe) in order to more accurately predict the behavior of genotypes under diverse environmental conditions.

Plants react to drought stress via a series of physiological, cellular, and molecular responses that converge in stress tolerance, avoidance, and/or escape (Delzon, 2015). While growth cessation has been identified as the earliest response to drought, stomatal closure is probably the most widely studied (Chaves *et al.*, 2010). Stomatal closure regulates plant water use and protects the xylem network from hydraulic failure (i.e. embolism) and branch dieback (Tyree and Sperry, 1989). Although there is a relationship between transpiration rate, leaf water potentials, and guard cell turgor (Brodribb *et al.*, 2003), there is still controversy regarding whether stomatal closure occurs well before, at, or even after the onset of embolism (Bartlett *et al.*, 2016). The leaf turgor loss point (π _TLP_) is defined as the water potential at which cells lose turgor (Brodribb *et al.*, 2003; Bartlett *et al.*, 2012) and has been considered a key physiological trait given its close association with stomatal closure and vulnerability to embolism (Brodribb *et al.*, 2003; Bartlett *et al.*, 2016; Martin-StPaul *et al.*, 2017; Zhu *et al.*, 2018). The π _TLP_ is estimated from the relationship between leaf water potential and leaf water volume known as the pressure–volume (p–v) curve and is related to other parameters correlated to drought tolerance such as the relative water content (RWC_TLP_), the osmotic potential at full hydration (π _0_), the elastic modulus of the cell wall (ɛ), and the tissue capacitance (C) (Bartlett *et al.*, 2012).

There is a well-known link between xylem embolism and mortality across tree species (Delzon and Cochard, 2014), and significant progress has been made in determining critical hydraulic thresholds for a number of woody species providing quantitative, ‘measurable’ indexes of their capacity to tolerate drought (Brodribb *et al.*, 2010; Urli *et al.*, 2013; Hammond *et al.*, 2019). Species vary in their vulnerability to embolism and are typically compared using the water potential inducing 50% loss of hydraulic conductance (P_50_) (Choat, 2013). Vulnerability may not be physiologically relevant under most non-extreme conditions where water status is modulated largely via stomatal regulation (Meinzer *et al.*, 2009). For example, most recent studies have shown that the hydraulic safety margin (HSM), defined as the difference between the water potential at stomatal closure (P_*gs*90_) and P_50_, may be more relevant in explaining plant mortality under drought than P_50_ alone (Anderegg *et al.*, 2016). Because the HSM integrates many important aspects of plant structure (e.g. anatomical features of the xylem and the cuticule) and physiology (e.g. stomatal conductance), its value across species is variable and related to the environment (Anderegg *et al.*, 2016). Additionally, other traits (e.g. osmotic adjustment, the capacitance of plant tissues, root system properties, etc.) could explain differences in xylem pressure changes and the rate at which critical thresholds for hydraulic failure are reached between plant species (Anderegg and Meinzer, 2015; Choat *et al.*, 2018). The recent Sur_Eau model developed by Martin-St. Paul *et al*. (2017) was successful in creating a framework for defining drought-induced mortality and could potentially be used to integrate multiple drought-related traits in predicting the time to hydraulic failure for different genotypes.

The objective of this study was to leverage multiple state-of-the-art phenotyping approaches to integrate a range of traits responsible for the drought response in three grapevine cultivars with contrasting water use behaviors, across the full range of water potentials leading to plant mortality. We hypothesized that the differences in drought behavior between cultivars involves multiple traits and is determined by the sequence and magnitude of the water potential thresholds for these traits. We quantified differences in the grapevine cultivars’ regulation of water use and linked these differences to leaf hydraulic traits such as π _TLP_ and P_50_, and the leaf HSM. To do so, we characterized each cultivar’s dynamic water use by continuously monitoring whole-plant transpiration and stomatal regulation using a greenhouse mini-lysimeter platform. Each cultivar’s corresponding leaf turgor loss point and its related parameters such as osmotic potential, modulus of elasticity, and leaf capacitance were determined through the construction of p–v curves. The leaf vulnerability to embolism was quantified in intact plants using the non-invasive optical technique (Brodribb *et al.*, 2016), allowing a comparison of HSMs between the cultivars. Finally, we used a soil–plant water transport model to explore the different associations between *E*_max_, P_*gs*90_, and P_50_ in determining the survival time until hydraulic failure during drought.

## Materials and methods

### Plant material

One-year-old plants of own rooted *Vitis vinifera* L. ‘Grenache’, ‘Syrah’, and ‘Semillon‘ from the INRA nursery (Villenave d’Ornon, France) were planted in 7 liter pots containing 1 kg of gravel and 5.5 kg of commercial potting medium (70% horticultural substrate and 30% sand). Plants were grown outside under well-watered conditions for ~2 months before the experiment started. The plants were drip irrigated with nutritive solution [NH_4_H_2_PO_4_ 0.1 mmol l^−1^; NH_4_NO_3_ 0.187 mmol l^−1^; KNO_3_ 0.255 mmol l^−1^; MgSO_4_ 0.025 mmol l^−1^; 0.002 mmol l^−1^ Fe, and oligo-elements (B, Zn, Mn, Cu, and Mo)] to avoid any deficiency during their development, and the surface of the pots were covered with a plastic bag to prevent water loss by soil evaporation.

### Mini-lysimeter phenotyping platform experiment

On 20 July, 15 plants from each cultivar were transferred to an automated mini-lysimeter greenhouse phenotyping platform where pots were continuously weighed on individual scales (CH15R11, OHAUS type CHAMP, Nänikon, Switzerland) and watered daily based on the plant weight loss by transpiration. The day before the experiment started, all the pots were irrigated up to their pot capacity weight and allowed to drain overnight. A dry-down experiment was imposed on 10 vines per cultivar by stopping irrigation on two different dates: 16 August on a set of five plants (*n*=5) per cultivar and 23 August on another set of five plants (each drying cycle lasted between 12 d and 15 d). A set of five well-watered vines per cultivar were kept as controls concomitantly with the water-stressed plants during each drying cycle. Air temperature, relative humidity, and radiation conditions were automatically monitored every 20 min at three different positions in the greenhouse. Air temperature was maintained approximately below 25 °C by the cooling system of the greenhouse to avoid any heat stress. The experimental design within the platform was completely randomized.

### Balance data analysis

The transpiration per leaf area (*E* in mmol m^−2^ s^−1^) was calculated as:

$$\begin{array}{l} E=\Delta_{w}/AL/MW_{w} \end{array}$$ (1)

where Δ _w_ is the change in weight within the considered period (g s^−1^), AL is the leaf area (m^2^), and MW_w_ the molecular weight of water (18 g mol^−1^).

The canopy stomatal conductance *G*_c_ (mmol m^−2^ s^−1^) was calculated using the simplification suggested by Monteith and Unsworth (1990):

$$\begin{array}{l} G_{c}=K_{G}\left(T\right)\times E/D \end{array}$$ (2)

Where *K*_G_(T) is the conductance coefficient in kPa m^3^ kg^−1^ (Ewers *et al.*, 2001) which accounts for temperature effects on the psychrometric constant, latent heat of vaporization, specific heat of air at constant pressure, and the density of air (Phillips and Oren 1998), *E* is the transpiration (mmol m^−2^ s^−1^), and D is the vapor pressure deficit (VPD; kPa) calculated from the temperature and relative humidity data, as indicated in the infrared gas analyzer manual WALZ GFS-3000.

The *G*_c_ values obtained were filtered by light in order to always have saturating values of radiation [photosynthetic photon flux density (PPDF) >800 μmol m^−2^ s^−1^].

The water status of the plants during the experiment was monitored by measuring the pre-dawn leaf water potential (ψ _PD_) on 3–4 plants per cultivar and treatment. Measurements were performed in a basal fully expanded leaf prior any light exposure (between 05.00 h and 06.00 h) every 2–3 d using a Scholander pressure chamber (Precis 2000, Gradignan, France).

The leaf area was estimated through the relationship obtained between the leaf midrib length and the leaf area (measured with a leaf area meter Model LI-3000, LI-COR, Lincoln, NE, USA) for each cultivar using ~200 leaves per cultivar. The leaf midrib length was measured weekly on all the leaves of each plant, and the respective total leaf area per plant was then calculated using the equations obtained.

Additional measurements of stomatal conductance were taken in fully expanded, mature leaves (8th–10th node) from all the cultivars and treatments. The measurements were taken at 11.00 h durng the 15 d of the experiment with a porometer (SC-1, Decagon Devices, WA, USA).

### Leaf turgor loss and minimum conductance

From the same set of plants, four plants per cultivar (*n*=4) were selected to construct the p–v curves by progressively drying leaves on a laboratory bench (‘bench dry method’; Sack and Pasquet-Kok, 2011) and measuring the ψ _leaf_ and leaf mass at determined intervals. The plants were well watered to pot capacity the previous day and drained overnight. In the morning (09.00–09.30 h), one healthy mature leaf (8th–10th leaf from the shoot base) was cut at the base with a razor blade, sealed in a plastic bag (Whirl-Pak), and its leaf water potential (ψ _leaf_) was measured with a Scholander pressure chamber (Precis 2000). The leaf mass was immediately registered in an analytical balance and the leaf was placed on a bench at room temperature (23 °C) to let it dehydrate until the next ψ _leaf_ and mass measurements. The time elapsed between measurements attempted to capture intervals of ψ _leaf_ of 0.2–0.3 MPa until at least five points were obtained beyond the point at which zero turgor was attained.

The p–v curves were constructed by plotting the inverse of leaf water potential (–1/ψ _leaf_) against the relative water content (RWC) which facilitated the determination of the turgor loss point as the point of transition between linear and non-linear portions (Supplementary Fig. S1 at *JXB* online; Tyree and Hammel, 1972). Leaf RWC was calculated as: RWC=(FW−DW)/(turgid weight−DW)×100. From the p–v curves, ψ _leaf_ at the turgor loss point (π _TLP_), osmotic potential at full turgor (π _0_), and modulus of elasticity (ε) were calculated according to Bartlett *et al.* (2012). Leaf capacitance (C_leaf_) was calculated from the change in volume per change in water potential at full turgor (C_FT_), and below the turgor loss point (C_TLP_).

In the same set of plants used to produce the p–v curves, minimum conductance (minimum water loss after stomatal closure) was determined in eight leaves from the three cultivars by using the ‘mass loss of detached leaves’ technique (Duursma *et al.*, 2019). The technique consists of measuring the leaf mass loss monitored over time as the leaf dries out. Leaves from well-watered plants were detached and suspended by their petiole (to allow the lamina to transpire from both sides) in a controlled chamber (Fitoclima 1200, Aralab, Portugal) set to a constant temperature of 25 °C and relative humidity of 45%. PPFD at the position of the samples was ~400±50 µmol m^−2^ s^−1^. The mass loss of the leaves was recorded every 5–10 min for the first hour and then every 15–20 min as long as the leaves dehydrated with a 0.0001 g resolution balance (Sartorius LE5201 Expert, Goettingen, Germany). A relationship between leaf mass and time allowed us to determine that initial water loss rates are high and after some time a constant flow is achieved. The minimum conductance (*g*_min_) is calculated by using the measured VPD according to the equation *E*=*g*_min_×D/P where D is the VPD in kPa and P is the atmospheric pressure expressed in mmol m^−2^ s^−1^ (Duursma *et al.*, 2019). The minimum conductance determined by this technique was used as a proxy of the ‘cuticular conductance’ as it has been recently observed in eight species that both conductances are comparable (Schuster *et al.*, 2017).

### Non-invasive optical determination of leaf vulnerability

Leaf embolism formation and propagation were evaluated in four individuals per cultivar (*n*=4) by monitoring changes in light transmission through the xylem (Brodribb *et al.*, 2016). Intact plants (well-watered) were placed in a room with controlled conditions at 26 °C and 50% relative humidity, and irrigation was cut off at the beginning of the scanning. For each plant, the abaxial side of an intact mature leaf (taken from the 8th–10th node), still attached to the parent vine, was fixed on a scanner (Perfection V800 Photo, EPSON, Suna, Japan) using a transparent glass and adhesive tape. The imaged area consisted of half of each leaf including the midrib, and the scanner magnification was set to give enough resolution of the midrib and at least eight major (second-order) veins. Brightness and contrast as well as leaf scanned area were adjusted to optimize visualization of embolisms and provide images not exceeding 9 Mbyte. Each leaf was automatically scanned every 5 min throughout plant dehydration using a computer automation software (AutoIt 3).

Simultaneous measurements of stem water potential (ψ _stem_) were made using psychrometers (ICT Internationale, Armidale, NSW, Australia) properly installed on the main stem adjacent to the scanned leaf. The ψ _stem_ values were automatically recorded every 30 min and the accuracy of the readings was confirmed by ψ _leaf_ measurements on basal leaves previously bagged with aluminum foil (2 h at least) using a Scholander pressure bomb (Precis 2000).

The stack of images captured at the end of the experiment comprised between 1800 and 2000 scans per leaf and they were analyzed using ImageJ software and following instructions from http://www.opensourceov.org. Briefly, total embolism was quantified by subtracting pixel differences between consecutive images (i.e. pixel values that did not change resulted in a value of zero). In these series, white pixels represented leaf embolism. Noise was removed using the ImageJ outlier removal, and pixel thresholding was used to extract embolism from any background noise remaining. The embolism area per image was calculated as the sum of non-zero pixels and expressed as cumulative embolisms, a percentage of total embolism area in the sequence.

Vulnerability curves corresponding to the percentage of embolized pixels (PEP) as a function of ψ _stem_ were fitted based on the following equation (Pammenter and Van der Willigen, 1998):

$$\begin{array}{l} PEP\;\;\;=\frac{100}{1+e^{\left(\;slp/25.\;\left(-P_{50}\right)\right)}} \end{array}$$ (3)

where P_50_ (MPa) is the ψ _stem_ value at which 50% of the xylem embolisms were observed and slp (% MPa^−1^) is the slope of the vulnerability curve at the inflexion point. The xylem pressure inducing the 12% (P_12_) and 88% (P_88_) of embolism loss of functionality were calculated as follows: P_12_=50/slp+P_50_ and P_88_= –50/slp + P_50_. One vulnerability curve was obtained per leaf per plant (*n*=4 per cultivar).

Finally, to visualize the dynamics of embolism spread through the leaf, spatio-temporal color maps of embolism formation were created for some of the samples by coloring the embolism area in each sequence using a color scale of ψ _stem_ over time.

### Model simulations of hydraulic traits

A soil–plant water transport model (Sur_Eau; Martin-StPaul *et al.*, 2017) was used to determine the predicted survival time until hydraulic failure under drought for each cultivar. A detailed explanation of the model is given in Martin-StPaul *et al.* (2017). Briefly, the plant is described as a series of variable symplasmic and apoplasmic hydraulic conductances and capacitances that determine water flows and water potential along the soil–plant–atmosphere continuum. The model computes the leaf transpiration, driven by leaf–air VPD, its regulation by stomatal closure, and thus the variation in soil water content. Beyond the point of stomatal closure, residual leaf transpiration is maintained, leading to plant dehydration and hydraulic failure under extreme water stress. Environmental conditions were either constant (T_air=25 °C, RH_air=50%, PAR=400 µmol m^−2^ s^−1^) or variable during the day (night: T_air=20 °C, RH_air=90%, PAR=0 µmol m^−2^ s^−1^; day as above). All plant parameters were identical for the three genotypes, except for the maximal stomatal conductance, the leaf osmotic potential at full turgor, the modulus of elasticity, the P_50_, and the stomatal response curve to leaf water potential that were adjusted to actual values measured on each genotypes. The simulations were performed at a time step of 0.01 s and stopped when 99.99 loss of hydraulic conductivity (PLC) was reached in the leaf apoplasm, thus defining the time to hydraulic failure.

### Statistical analyses

Statistical analyses and fit were performed using R software (http://www.R-project.org) and GraphPad Prism version 7.00 for Windows (GraphPad Software, La Jolla, CA, USA).

To extrapolate water potentials obtained during the dry-down experiment at the lysimeter platform, ψ _PD_ was calculated from RWC using a Campbell (1974) equation modified by Van Genuchten (1980) (Charrier *et al.*, 2018; Supplementary Fig. S2):

$$\begin{array}{l} \psi_{PD}=a\times {\left(RWC\right)}^{b}+\psi_{e} \end{array}$$ (4)

where ψ _e_ is the soil water potential at the air entry point.

The soil RWC of each pot was calculated as RWC=(actual weight−DW)/(saturated weight−DW). The saturated weight corresponded to the total pot weight registered after watering the plants to pot (or field) capacity at the end of the afternoon and drained overnight. The actual weight corresponded to the weight registered by the balances during the dry-down experiment, and the dry weight corresponded to the weight of a pot after placing in an oven for 48 h at 60 °C. The weight of the soil substrate was obtained by subtracting the weight of the pot and draining gravel (which was standardized between pots).

Stomatal conductance (*G*_c_), depending on pre-dawn leaf water potential, was fit according to the following sigmoid function:

$$\begin{array}{l} G_{c}={\frac{g_{sm}}{1+{e^{slp.}}^{-Pg_{s50}}}}_{\;} \end{array}$$ (5)

where *g*_*sm*_ corresponds to maximal stomatal conductance at ψ=0, slp, the sensitivity to decreasing water potential, and P_*gs*50_ the water potential inducing 50% stomatal closure.

Linear regressions between *G*_c_ and VPD were calculated for each cultivar, and the slopes and intercepts were compared at *P*<0.05 using analysis of covariance (ANCOVA) in R. For non-linear regressions (*G*_c_ and *E* versus ψ _PD_), the Akaike information criterion (AICc) method was used to compare different fits.

Difference in P_50_, P_12_, P_88_, and slp, and p–v results were tested with one-way ANOVA and post-hoc Tukey HSD test (<0.05). The HSM was calculated for each cultivar as the difference between the water potential at P_*G*c90_ and the water potential at stem P_50_.

## Results

### Water use under non-limiting water conditions

**Fig. 1.**
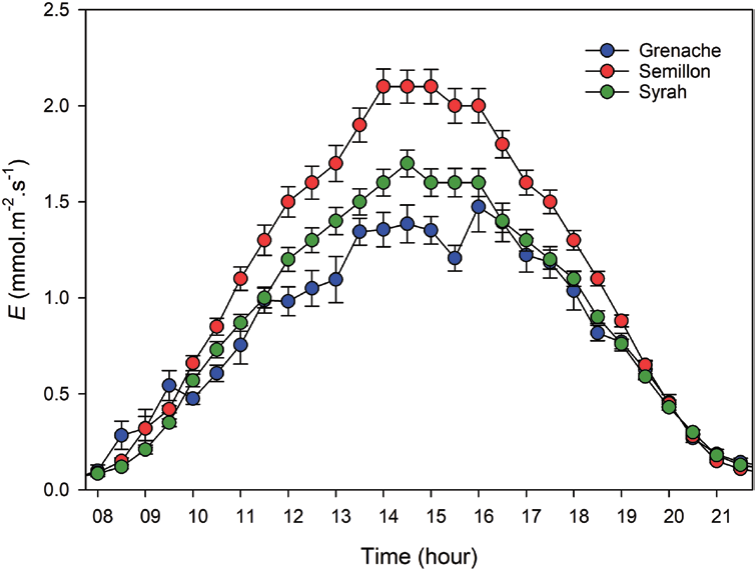
Daytime transpiration rate measured at the whole-plant scale and expressed per leaf area in well-watered plants of Grenache, Semillon, and Syrah. Each point is the hourly mean across 15 d of the experiment ±SE (*n*=50–60).

**Fig. 2.**
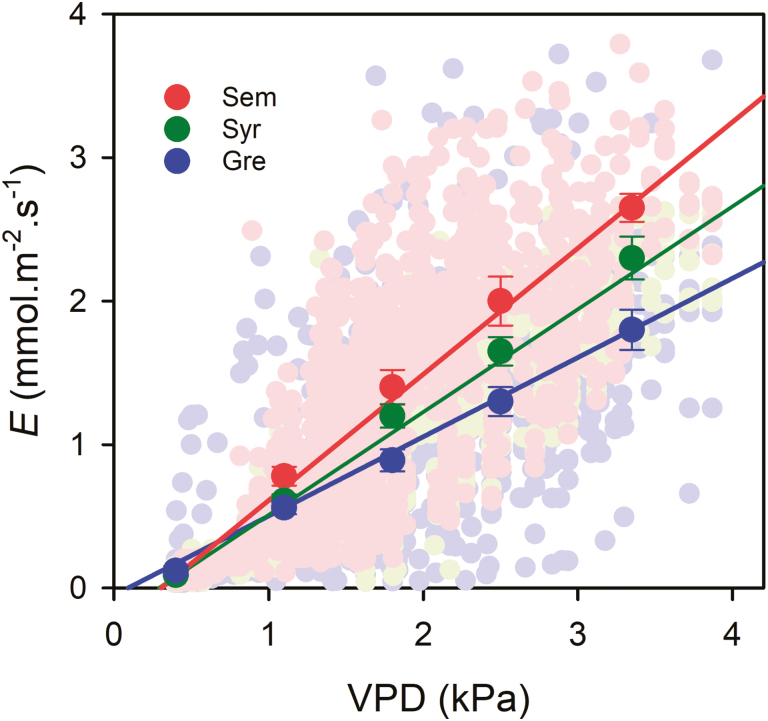
Daytime (08.00 h–20.00 h) plant transpiration rates (*E*) depending on vapor pressure deficit (VPD) in three grapevine cultivars (Grenache, Semillon, and Syrah) under well-watered conditions (ψ _PD_ greater than –0.5 MPa). Significant differences were found for the slope (*P*<0.0001) of the linear regression lines (Grenache, *E*=0.5525VPD-0.0503; Semillon, *E*=0.8794VPD-0.2689; Syrah, *E*=0.7171VPD-0.2073). Light colored dots are single values registered across 15 d of the experiment and dark colored dots represent the mean of 0.5 kPa ranges of VPD ±SE.

Under well-watered conditions, diurnal rates of transpiration (*E*) in all plants increased from minimum values overnight to maximum rates (*E*_max_) at ~14 h and were maintained for several hours (Fig. 1). Significant differences were observed in *E*_max_ between cultivars, with Semillon showing the highest rate, 2.1 mmol H_2_O m^−2^ s^−1^, and Grenache the lowest, 1.4 mmol H_2_O m^−2^ s^−1^. It is important to mention that the total leaf area was slightly lower in Semillon (~17% lower than both Syrah and Grenache); however, these differences did not result in different rates of the dry-down between cultivars (not shown), which was mostly explained by differences in *E*_max_. The distribution of the data also showed that Semillon had a higher frequency of high rates of *E* than Grenache, with Syrah being intermediate (Supplementary Fig. S3). Under drought conditions, *E* was significantly reduced during the day for all cultivars. The highest values of *E* of these plants were 0.13 mmol H_2_O m^−2^ s^−1^ at midday without differences between cultivars. An examination of the response of *E* to VPD in well-watered plants (ψ _PD_ greater than –0.5 MPa) revealed a linear positive relationship for the three cultivars (Fig. 2). Transpiration of the three cultivars increased with increasing VPD across a range of 0.36–3.86 kPa; however, a clear distinction among them was observed, where Semillon and Grenache showed the highest and lowest slopes, respectively.

### Regulation of water use under water deficit

**Fig. 3.**
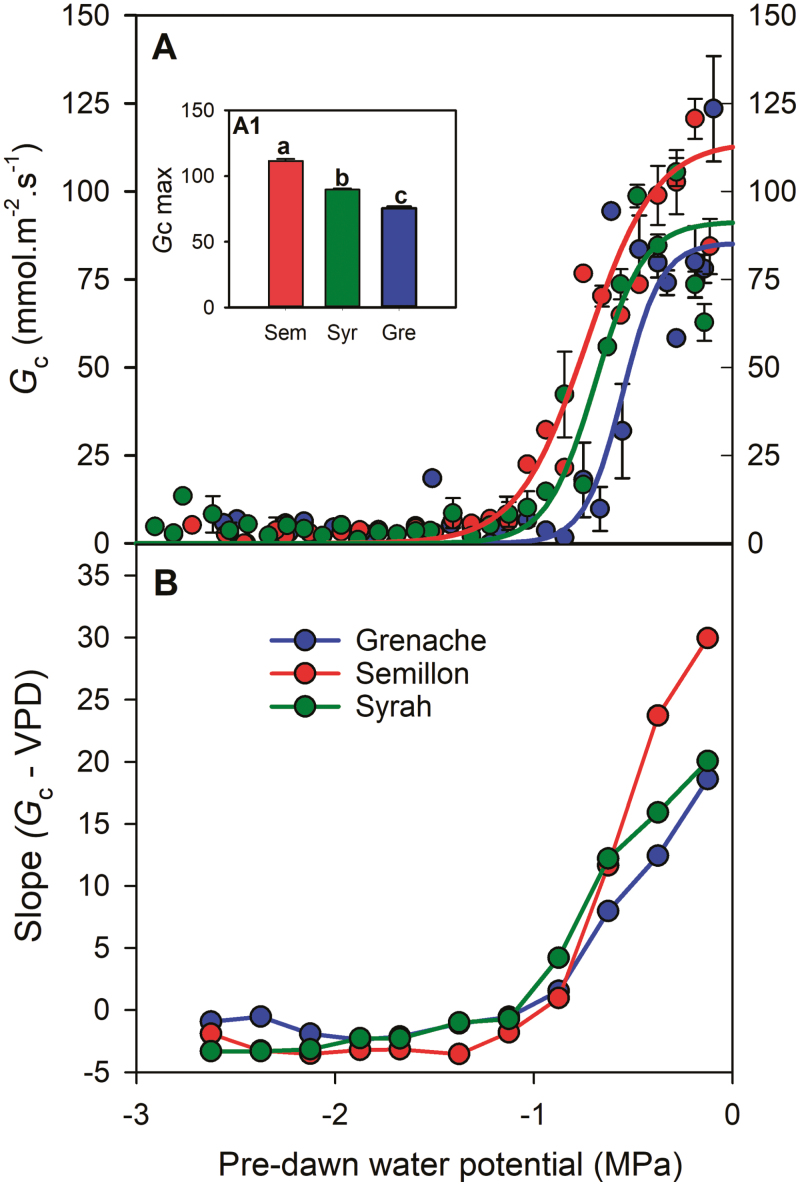
(A) Stomatal conductance (*G*_c_) at the whole-plant scale (expressed per leaf area) measured between 10.30 h and 15.00 h, and (B) the slope of *G*_c_ versus VPD [Slope (*G*_c_–VPD)] as a function of pre-dawn water potential (ψ _PD_) in three grapevine cultivars (Grenache, Semillon, and Syrah). Inset figure (A1), mean stomatal conductance registered between 10.30 h and 15.00 h across 15 d of the experiment under well-watered conditions (ψ _PD_ greater than –0.5 MPa). In (A), each point represents the mean ±SE of 0.1 MPa classes. Lines represent the best fit using sigmoid functions for each cultivar.

The three cultivars exhibited a sigmoidal relationship between *G*_c_ and ψ _PD_ (Fig. 3A) where *G*_c_ declines from maximum values in the range of ψ _PD_ between 0 and –0.5 MPa to its minimum at less than –1.2 MPa. Differences in the maximum level of *G*_c_ (*G*_c_ max) mirrored differences in *E*_max_ (Fig. 3A, inset), with Semillon exhibiting the highest *G*_c_ max, followed by Syrah and then Grenache. The sensitivity of stomata to drought varied among cultivars, with water potential at 90% of stomatal closure (P_*G*c90_, determined from *G*_c_) of –0.80 MPa in Grenache, –1.00 MPa in Syrah, and –1.20 MPa in Semillon (Fig. 3A). The sensitivity of *G*_c_ to VPD (the lower the slope of the relationship *G*_c_ versus VPD the more sensitive) was greatest for Grenache and lowest for Semillon but only under well-watered conditions (Fig. 3B).

The stomatal conductance values measured with the porometer were higher (around double) than those calculated from the balances data; however, the differences between cultivars were maintained in a similar way as in Fig. 1 (not shown). Also, the water potential at 90% of stomatal closure (P_*gs*90_, determined from leaf-level *g*_s_) calculated with the porometer did not differ significantly as compared with that from the balances (Grenache= –0.78MPa, Semillon= –1.35, and Syrah=1.32 MPa).

### Leaf hydraulic traits

Different parameters were compared between cultivars from the analysis of p–v curves (Table 1; Supplementary Fig. S1). Only π _TLP_ and π _0_ differed between cultivars, where Semillon showed a significantly more negative π _TLP_ (–1.92 MPa) and π _0_ (–1.55 MPa) than those of Syrah and Grenache.

**Table 1.** Pressure–volume (p–v) parameters derived from p–v analysis curves in three grapevine genotypes

Genotype	SWC	π _o_ (MPa)	π _TLP_ (MPa)	RWC_TLP_ (%)	ɛ (MPa)	C_FT_ (MPa^−1^)	C_TLP_ (MPa^−1^)
Grenache	2.63±0.23	–1.18±0.07 b	–1.38±0.06 b	89.41±2.32	12.97±2.45	0.07±0.01	0.33±0.07
Semillon	2.32±0.06	–1.55±0.03 a	–1.92±0.10 a	84.58±1.80	11.8±1.49	0.08±0.01	0.32±0.06
Syrah	2.18±0.09	–1.34±0.03 b	–1.50±0.04 b	89.44±1.59	14.16±1.54	0.07±0.01	0.42±0.16
	ns	*	*	ns	ns	ns	ns

Values are the average ±SE (*n*=4). Significance: * and ns indicate significance at *P*≤0.05 and not significant, respectively.

Means within columns followed by different letters differ significantly at *P*≤0.05 by Fisher’s multiple range test.

SWC, saturated water content; π _o_, osmotic potential at full turgor; π _TLP_, turgor loss point; RWC_TLP_, relative water content at turgor loss point; ɛ, modulus of elasticity; C_FT_, capacitance at full turgor. C_TLP_, capacitance at turgor loss point.

The leaf minimum conductance was not different between cultivars, with presented mean values of ~9.5 mmol m^−2^ s^−1^ (Supplementary Fig. S4).

**Fig. 4.**
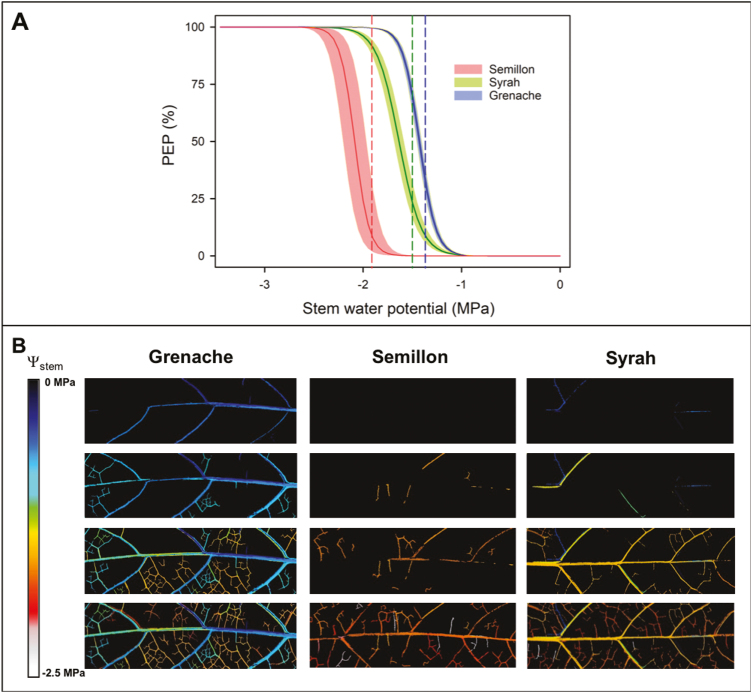
(A) Optical vulnerability curves expressed as the percentage of embolized pixels (PEP) as a function of stem water potential in three grapevine cultivars (Grenache, Semillon, and Syrah). The solid dark line and shaded bands represent the mean observed embolism ±SE for each cultivar. The Pammenter model was first fitted per sample (*n*=4) per cultivar before calculating the mean P_50_ and slope. The colored dotted lines indicate the leaf turgor loss point (π _TLP_) for each cultivar (B) Representation of leaf embolism spread during the progress of dehydration in three grapevine cultivars (Grenache, Semillon, and Syrah). Embolisms are colored according to the water potential at which they occurred (color scale shown).

Using the optical technique (Brodribb *et al.*, 2016), leaf vulnerability to embolism was quantified in intact plants (Fig. 4). The water potential at which 12% of embolisms occurred (P_12_) indicated considerable variation among cultivars. Values of P_12_ were estimated at –1.26 MPa for Grenache, –1.41 MPa for Syrah, and –1.94 MPa for Semillon. The leaf vulnerability curves constructed from the data obtained followed a sigmoidal function for all genotypes and showed variability between individual leaves, mainly for Semillon (Fig. 4A). The water potentials at which embolism occurred was also significantly different between cultivars, where Grenache showed embolism earlier (P_50_ at –1.43 MPa), followed by Syrah (P_50_ at –1.65 MPa) and Semillon (P_50_ at –2.08 MPa). Finally, the water potentials at which 88% of the embolism occurred (P_88_) were observed at –1.8 MPa for Grenache, –2.24 MPa for Semillon, and –1.86 MPa for Syrah.

### The hydraulic safety margin and trait relationships

**Fig. 5.**
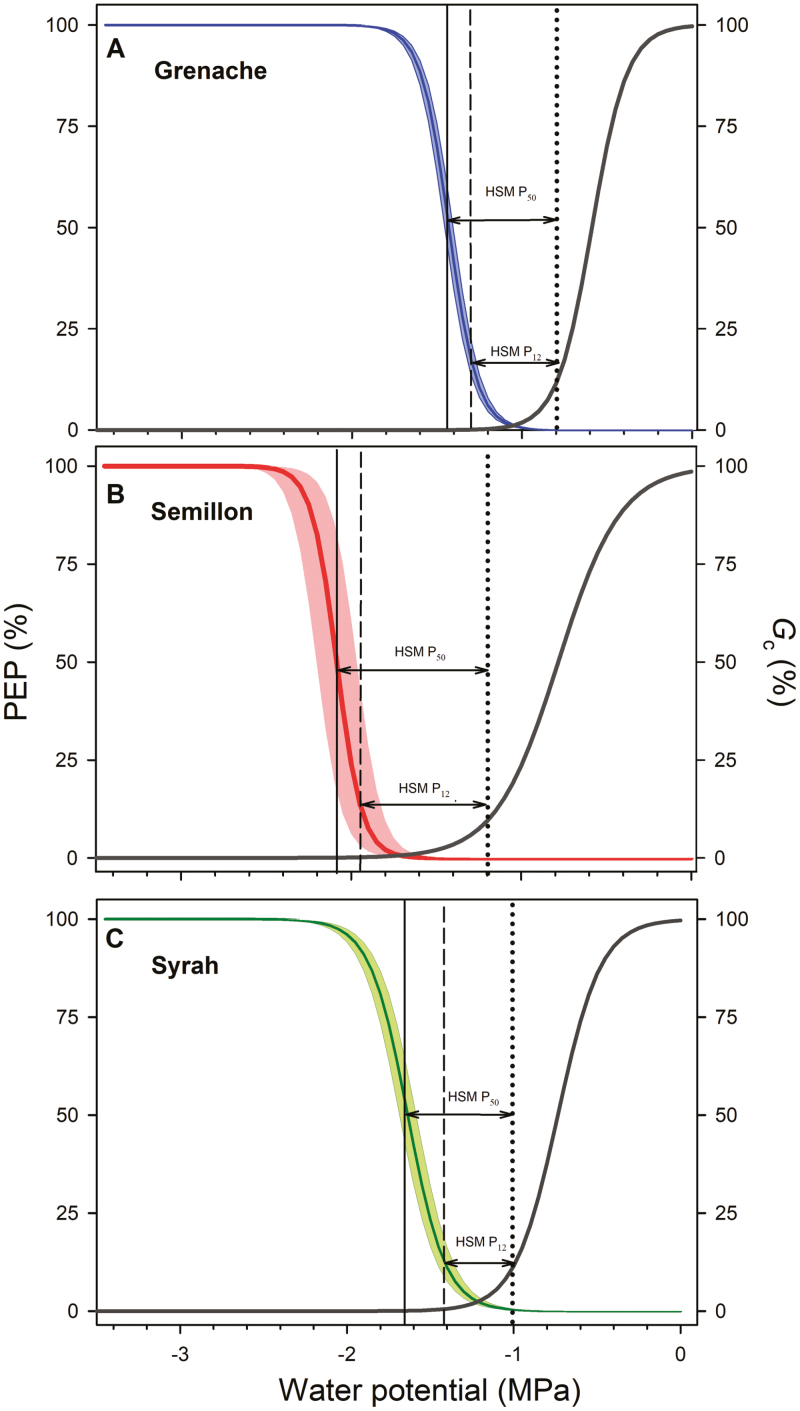
Optical hydraulic vulnerability curves (PEP %) and stomatal conductance (*G*_c_ %) as a response of the organ (stem for PEP % and leaf for *G*_c_) water potential in three grapevine cultivars, (A) Greanche, (B) Semillon, and (C) Syrah. The P_*G*c90_, P_50_, and P_88_ are indicated by a dotted, dashed, and solid black line, respectively. The hydraulic safety margins at P_50_ (HSM P_50_) and at P_12_ (HSM P_12_) are indicated by arrows.

The difference between P_*G*c90_ and P_12_ were greatest for Semillon (0.74 MPa), intermediate for Grenache (0.46 MPa), and lowest for Syrah (0.41 MPa; Fig. 5). Similarly, differences betwseen P_*G*c90_ and P_50_ (HSM P_50_) were greater in Semillon (0.88 MPa) followed by Syrah (0.65 MPa), and finally Grenache (0.63 MPa).

**Fig. 6.**
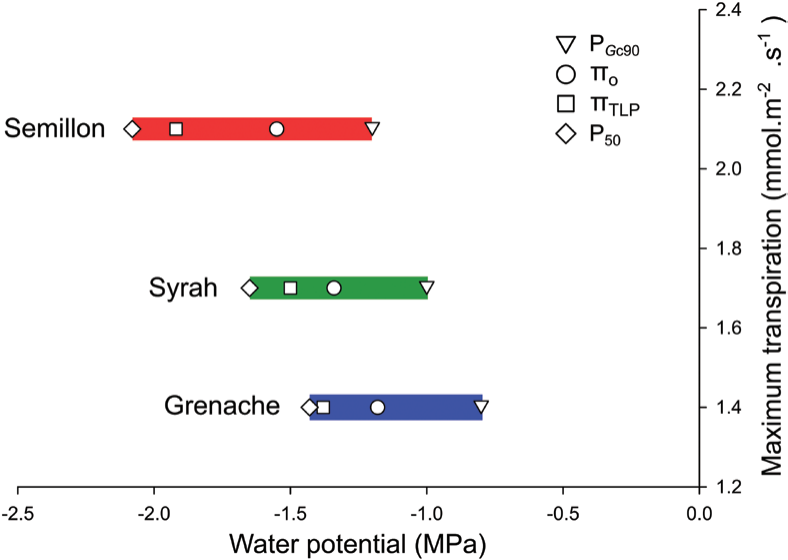
Relationships between the maximum transpiration rate for the three cultivars under well-watered conditions and the leaf-level hydraulic traits P_*G*c90_, π _0_, π _TLP_, and P_50_. Bars correspond to the HSM P_50_. Pearson correlations between all parameters can be found in Supplementarty Fig. S5.

Many of the hydraulic traits quantified in the current study were well correlated with each other across the three cultivars (Supplementary Fig. S5). A cultivar’s *E*_max_ under well-watered conditions was strongly correlated with several traits including P_*G*c90_, π _0_, π _TLP_, and P_50_ (Fig. 6). A cultivar’s P_50_ was strongly correlated with P_*G*c90_, π _0_, π _TLP_, C_FT_, and RWC_TLP_ (Supplementary Fig. S5). Finally, a cultivar’s HSM P_50_ was strongly correlated with several traits, including π _TLP_.

### Simulating the time of survival under drought

**Fig. 7.**
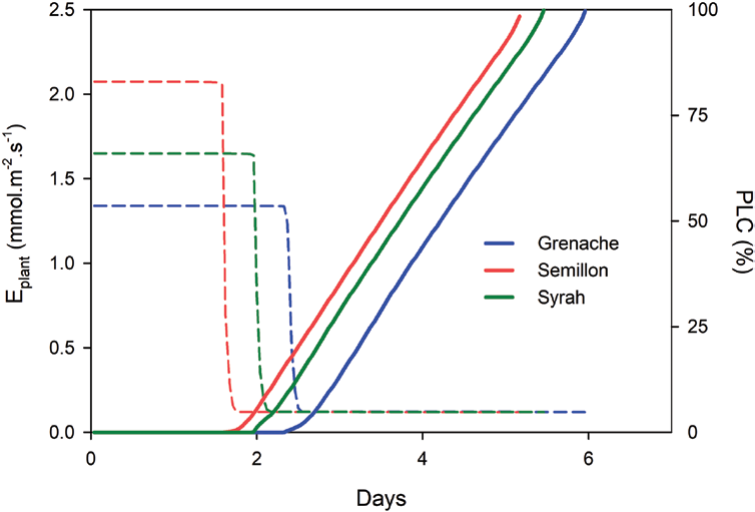
Simulated declining of maximum water use (*E*_plant_; dotted lines) and increasing loss of hydraulic conductivity (PLC%; solid lines) over time (days) under identical environmental conditions during the progression of drought in three grapevine cultivars (Grenache, Semillon, and Syrah) using the Sur_Eau model (Martin-StPaul *et al.*, 2017).

Drought simulations performed with the Sur_Eau model using the hydraulic traits empirically determined in this study showed that when the three different cultivars are placed under identical environmental conditions, the maximum water use (*E*_plant_) decreased earlier or later depending on the cultivar. For instance, *E*_plant_ decreased significantly after 1.5 d in Semillon, 2.0 d in Syrah, and 2.5 d in Grenache (Fig. 7). Also, the predicted time in this ‘generic scenario’ needed to reach P_50_ (used here to approximate mortality) was also different across cultivars where Semillon reached P_50_ in 3.5 d, Syrah in 3.75 d, and Grenache in 4.2 d. When including variable climatic conditions (day/night simulations) in the model, the predicted time needed to reach P_50_ was higher for all the cultivars, being 7.3 d in Semillon, 7.6 d in Syrah, and 8.7 d in Grenache (Supplementary Fig. S6).

## Discussion

In this study, we integrated hydraulic traits to explain the drought responses of three contrasting grapevine varieties across a wide range of soil water deficit and VPD conditions. Cultivars differed in multiple traits examined, including *E*_max_, stomatal regulation, π _TLP_, π _0_, and leaf vulnerability to embolism (P_50_). Despite these differences, all cultivars closed their stomata prior to leaf embolism formation. The maximum water use given by *E*_max_ was strongly correlated with other drought traits (e.g. P_*G*c90_, π _TLP_, and leaf P_50_) and thus the higher *E* in Semillon corresponded to a less vulnerable leaf exhibiting a more negative π _TLP_ and leaf P_50_. There was substantial variation of the cultivars’ HSMs, and model simulations suggest that despite Grenache’s narrower HSM, its more conservative water use results in it having a longer time to hydraulic failure than the other cultivars.

### Maximum transpiration

Monitoring real-time whole-plant water use in the mini-lysimeter phenotyping platform revealed differences in the water use behavior between the three cultivars. Under well-watered conditions, Semillon had greater water use over a wide range of VPD conditions (Figs 1, 2). It has been reported that Semillon displays higher rates of daytime (and night-time) transpiration under field conditions when compared with other grapevine cultivars (Rogiers *et al.*, 2009). Under well-watered conditions and saturating values of radiation (PPDF >800 μmol m^−2^ s^−1^), *E* responded linearly to VPD in all genotypes since VPD is a main driver of transpiration (Oren *et al.*, 1999; Schultz and Stoll, 2010; Rogiers *et al*., 2011; Devi and Reddy, 2018). A linear relationship between *E* and VPD was also observed across a diverse set of species under well-watered conditions (Oren *et al.*, 1999). Semillon also exhibited a low sensitivity of *G*_c_ to VPD under well-watered conditions. Grapevine *G*_c_ has been shown to be relatively insensitive to VPD under well-watered conditions (Rogiers *et al*., 2011; Charrier *et al.*, 2018), and grapevines have been shown to maintain high levels of *E* and *G*_c_ even at very high temperatures (>40 °C) when supplied with ample irrigation (Greer, 2012).

### Stomatal regulation

A decline of stomatal conductance was observed with increasing soil water deficit in all cultivars (Fig. 3A). However, Grenache exhibited a more rapid decline and a higher sensitivity of *G*_c_ to increasing VPD. These results are consistent with previous studies that suggest that Grenache regulates ψ _leaf_ more ‘conservatively’ than other varieties, exhibiting an isohydric behavior (Schultz, 2003). However, differences in the stomatal sensitivity to VPD between cultivars occurred only within a narrow range of pre-dawn water potentials (greater than –1 MPa), below which all cultivars displayed a similar response (Fig. 3B). Recent investigations on different grapevine cultivars and rootstocks also observed that differences between cultivars in the regulation of *G*_c_ were only present under well-watered and mild stress conditions (greater than –0.8 MPa) (Peccoux *et al*., 2017; Charrier *et al.*, 2018). The complexity of the relationships between stomatal regulation, soil water potential, and VPD makes assigning distinct water use behaviors (e.g. iso/anisohydric) difficult (Hochberg *et al.*, 2018) and so we sought to integrate these behaviors with other hydraulic traits.

In this study, *G*_c_ was calculated from whole-plant transpiration using Equation 2 which uses ambient VPD as the driving force for the calculation. This allows for the use of a much greater amount of data recorded in real time by the mini-lysimeter platform, which is critical for larger scale phenotyping and more robust statistical analysis. However, the use of ambient temperature and not leaf temperature could bias our results, and studies have shown that in complex grapevine canopies, the degree of coupling between the canopy and atmosphere can be complex (Buckley *et al.*, 2014). Therefore, we also carried out leaf-level stomatal conductance measurements which were strongly correlated with values of *G*_c_ (*r*^2^=0.79, *P*<0.0001, data not shown), and our values of P_*gs*90_ calculated from leaf-level measurements did not differ significantly from P_*G*c90_. Additionally, previous studies from our laboratory have shown a strong correlation between stomatal conductance measurements made at the leaf- and whole-plant levels (Charrier *et al.*, 2018) in our experimental context. Future studies could aim at using more robust methods of extrapolating stomatal conductance from whole-plant water use data.

### Leaf-level hydraulic traits

In our study, the more negative P_*G*c90_ values observed in Syrah and Semillon could allow them to operate over a wider range of water potentials, and this is likely to be due in part to their lower osmotic potential at full hydration (π _0_) and leaf turgor loss (π _TLP_) relative to Grenache (Table 1). It has been suggested that the π _TLP_ is related to a plant’s ability to ‘tolerate’ drought rather than to avoid it by closing the stomata or shedding leaves (Brodribb and Holbrook, 2003). Other p–v parameters have also been proposed as strong predictors of drought tolerance, such as the modulus of elasticity (Niinemets, 2001; Brodribb and Holbrook, 2003; Read *et al.*, 2006). An interesting meta-analysis conducted across many species concluded that π _TLP_ showed a strong association with water availability within and across habitats and that π _0_ and π _TLP_ were the only two parameters that clearly delimited species associated with wet and dry habitats (Bartlett *et al.*, 2012). The same study showed that variations in π _TLP_ among and within species were mainly driven by osmotic (π _0_) rather than elastic adjustments to maintain RWC_TLP_ and prevent cell dehydration. In our study, Semillon also showed a significantly lower π _o_ than Syrah and Grenache, suggesting that osmotic adjustment was the main driver of its more negative turgor loss point. The ability to generate solutes or tolerate an increased symplastic solute concentration has been observed to differ between species (Zhang *et al.*, 1999). Because producing solutes generates a metabolic cost, some species will rely (or are more plastic) on other physiological traits to survive drought such as root morphology, water use efficiency, and xylem cavitation resistance (Vandeleur *et al.*, 2009; Choat *et al.*, 2010; Bartlett *et al.*, 2012; Gambetta *et al.*, 2013; Barrios-Masias *et al.*, 2015). Based on these observations, it is reasonable to conclude that the ability of Semillon to maintain cell turgor at a lower ψ _leaf_ allowed this cultivar to operate across a wider range of soil water deficit and VPD conditions than Syrah and Grenache.

### Leaf vulnerability to embolism

Leaves of the different cultivars varied significantly in their vulnerability to embolism (Fig. 4). Only a few studies to date (e.g. Martorell *et al.*, 2015) have compared the leaf hydraulic vulnerability between different grapevine genotypes in the same study. The range of leaf P_50_ values (ranging from –1.4 MPa to –2 MPa) in the current study was consistent with the leaf P_50_ values in Syrah (–1.5 MPa; Hochberg *et al.*, 2016) and Chardonnay (–1.5 MPa to –2.0 MPa; Hochberg, 2017*b*). Interestingly, all cultivars experienced enough stomatal closure before any leaf embolisms were observed (Fig. 5). Our results argue against the hypothesis that the induction of embolism could act as a signal for stomatal closure (Nardini and Salleo, 2000) and support the recent findings that significant embolism occurs after stomatal closure under prolonged drought conditions (Hochberg *et al.*, 2016; Martin-StPaul *et al.*, 2017; Creek *et al*., 2020).

In the current study stomatal regulation is related to pre-dawn water potentials (Fig. 3A) while vulnerability is related to stem water potentials (Fig. 4A). It would have been more appropriate to use the stem water potentials at the time of stomatal conductance since there is a possibility that, relative to the vulnerability thresholds, P_*G*c90_ would be slightly overestimated, making the resulting HSM overestimated as well. However, this is unlikely since at P_*G*c90_, pre-dawn and midday water potentials collapse to the same value (i.e. ψ _PD_~ψ _Stem_) which has been shown before for two of the same cultivars (Grenache and Syrah) within the same experimental system (Charrier *et al.*, 2018).

### Linking traits: from *E*_max_, to stomatal closure, to leaf vulnerability

In our study *E*_max_ under well-watered conditions was strongly correlated with numerous leaf-level hydraulic traits including P_*G*c90_, π _0_, π _TLP_, and P_50_ (Fig. 6). This suggests that as maximum water use increases, leaf hydraulic traits are well integrated, preparing the leaf to operate across a wider range of water potentials. Simonin *et al*. (2014) demonstrated that across several forest tree species, leaf hydraulic conductance increases with increasing *E*. In that study, although the increase in leaf hydraulic conductance buffered variation in ψ _leaf_, increasing *E* led to more negative ψ _leaf_, increasing the range of ψ _leaf_ experienced by the plants. Similarly, a close relationship between leaf turgor loss, loss of hydraulic conductance, and stomatal closure was observed in different tree species, suggesting that stomatal closure is coordinated with leaf hydraulic conductance (Brodribb and Holbrook, 2003). In the current study, other leaf-level hydraulic traits were also well coordinated with each other (Supplementary Fig. S5), consistent with previous studies (Bartlett *et al.*, 2016). This raises the possibility that leaf hydraulic traits are largely interdependent and raises questions about the extent to which these traits can be disentangled (Reich, 2014).

Complete stomatal closure occurred prior to the π _TLP_ for all cultivars, indicating that the stomatal response to ψ _leaf_ occurs as mesophyll cell turgor declines (Fig. 5; Table 1). Similar results have been found previously for the grapevine cultivar Merlot (Hochberg *et al.*, 2017*a*). A recent study in a number of species of different biomes indicated that the water potential causing stomatal closure is closely related to π _TLP_ in most of the species (Martin-StPaul *et al.*, 2017). However, studies across other species have shown that the relationship between stomatal closure and π _TLP_ can vary, with some species closing stomata coincident with, and others prior to, the π _TLP_ (e.g. Brodribb and Holbrook, 2003; Guyot *et al.*, 2012). Such uncoupling between stomatal closure and π _TLP_ is supported by previous evidence showing that guard cells can act independently from the rest of the leaf (Mott and Franks, 2001). Stomatal closure prior to π _TLP_ is considered a drought tolerance mechanism enabling a plant to survive on stored water (which would be slowly lost considering a low minimal conductance) after stomatal closure (Guyot *et al.*, 2012; Delzon, 2015; Duursma *et al.*, 2019).

A cultivar’s π _TLP_ was closely related to the onset of leaf xylem embolism (Fig. 4). Although we did not quantify leaf mortality in the current study we carried out a re-analysis with the results obtained by Charrier *et al.* (2018) in order to examine the relationship between leaf PLC and mortality for Grenache and Syrah. In both cultivars, water potential values corresponding to high levels of leaf PLC (>50%, or even 88%) in the current study corresponded to low levels of leaf mortality in the study of Charrier *et al.* (2018), from 5% to 10 % in Grenache and from 20% to 30% in Syrah. This supports the hypothesis that significant levels of leaf embolism precede leaf mortality in grapevine (Hochberg *et al.*, 2016) and other angiosperms (Urli *et al.*, 2013; Choat *et al.*, 2018).

Because the HSM can be used to predict tree mortality rates in many forest biomes, it has been used to evaluate the degree of conservatism in plant hydraulic strategies to drought (Brodribb *et al.*, 2010; McDowell *et al.*, 2011; Choat *et al.*, 2013). In our study, the cultivar that had the most negative P_50_ (Semillon) also exhibited the largest HSM when compared with the other cultivars (Fig. 5). Despite this, there appears to be no systematic relationship between P_50_ and the size of the HSM. Grenache, for example, with the least negative P_50_ still maintained a significant HSM because of its less negative P_*gs*90_. The results of the simulations performed with the model Sur_Eau showed that the most ‘conservative’ cultivar (Grenache) could be considered the most drought tolerant because the time needed to reach P_50_ is greater than for the other cultivars (Fig. 7; Supplementary Fig. S6). This is intriguing given that Grenache is widely considered a drought-tolerant cultivar that is selectively chosen for cultivation in hot, dry wine regions. This result also illustrates that the timing of hydraulic failure does not correlate directly with the size of the HSM and instead is a more complex process where other traits such as stomatal regulation and minimal conductance are involved.

Differences in the size of the leaf HSM between cultivars could be brought about by differences in P_*G*c90_ and/or leaf P_50_. In the current study, P_*G*c90_ and P_50_ (and π _TLP_) were well correlated with each other, and with *E*_max_ (discussed above), with greater *E*_max_ corresponding to a more negative P_*G*c90_, π _TLP_, and P_50_ (Fig. 6). Recent investigations demonstrated that across diverse species, π _TLP_ was positively correlated with leaf minimum water potential and vulnerability to embolism (i.e. the less negative the π _TLP_, the greater the HSM), although the variation within this relationship was extremely large (Zhu *et al.*, 2018). This correlation does not hold between the grapevine cultivars examined here. For example, Semillon exhibited the most negative P_50_ and π _TLP_, and the largest safety margin (Figs 4, 5). These results support the idea that although water use and leaf hydraulic traits are often correlated, these relationships are variable even within a single species such as grapevine (Brodribb *et al.*, 2003; Choat *et al.*, 2018).

The exact mechanisms leading to correlations between traits and observed differences in the HSM remain unknown. Once the stomata close, the plant relies on the internal stored water for survival (Blackman *et al.*, 2016), and its depletion after stomatal closure depends mostly on the minimum conductance, *g*_min_ (Duursma *et al.*, 2019). This is the first study to quantify *g*_min_ in grapevine, and we did not find differences in *g*_min_ between the three cultivars (Supplementary Fig. S4), with the absolute values being similar to those of several other crop species (Duursma *et al.*, 2019). Other traits that could potentially influence the HSM could include leaf capacitance, leaf shrinkage (Scoffoni *et al.*, 2014), transpiration area variation (i.e. leaf shedding), and total leaf water storage (Choat *et al.*, 2018). Processes that occur after leaf stomatal closure are surely critical and deserve more attention.

### Conclusions

This is among one of a few studies on grapevine that has attempted to integrate multiple drought behavior traits. All cultivars closed stomata prior to reaching water potential values that would cause leaf hydraulic failure. Traits that allow a cultivar to tolerate lower ψ _leaf_, such as π _0_, π _TLP_, and P_50_, were tightly correlated with a cultivar’s maximum water use under well-watered conditions. Despite these correlations, there was substantial variability in the HSM between cultivars, suggesting that the relationships between leaf hydraulic traits are labile even within a single species such as grapevine. The results of this study demonstrate that the relationships between traits such as stomatal regulation, leaf embolism thresholds, and survival time under drought are complex and interactive, stressing the importance of integrating multiple physiological traits in characterizing drought tolerance.

## Supplementary data

Supplementary data are available at *JXB* online.

Fig. S1. Pressure–volume curves plotted as –1/ψ versus RWC for each cultivar.

Fig. S2. Correlation between pre-dawn water potential and soil relative water content.

Fig. S3. Variability of diurnal plant transpiration. (A) Boxplot and (B) histogram.

Fig. S4. Minimum leaf conductance for each cultivar.

Fig. S5. Pearson correlations between the measured hydraulic traits.

Fig. S6. Simulation with the Sur_Eau model under variable climatic conditions.

eraa186_suppl_Supplementary_Figures
