# Supplementary material for: The sequence and thresholds of leaf hydraulic traits underlying grapevine varietal differences in drought tolerance
Source: J Exp Bot. 2020 Apr 11;71(14):4333–44. doi: 10.1093/jxb/eraa186 (PMC7337184; doi:10.1093/jxb/eraa186)

Fig. S1 Pressure-volume curves plotted as -1/Ψ versus RWC to facilitate parameter estimation in three grapevine cultivars (A) Grenache, (B) Semillon, (C) Syrah.

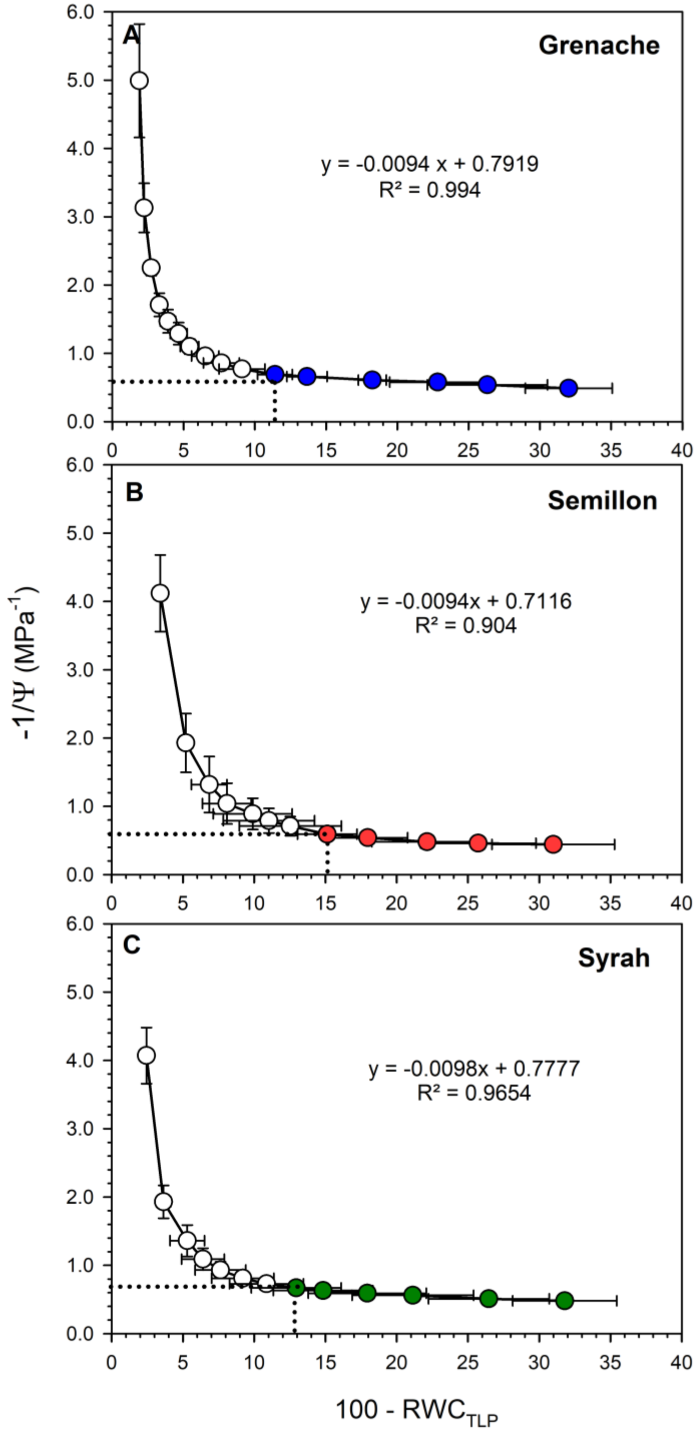

Fig. S2 Correlation between pre-dawn water potential and soil relative water content in three grapevine cultivars (Grenache, Semillon and Syrah) during the mini-lysimeter greenhouse experiment. Different colored lines represent the fit according to Campbell-van Genuchten equation ($\Psi_{PD}=a\cdot(RWC)^{-b}+\Psi_e$; where Ψ_e is the soil water potential at the air entry point). Significant differences ($p<0.0001$) were observed in the fittings between Semillon and the other two cultivars that had a similar fitting.

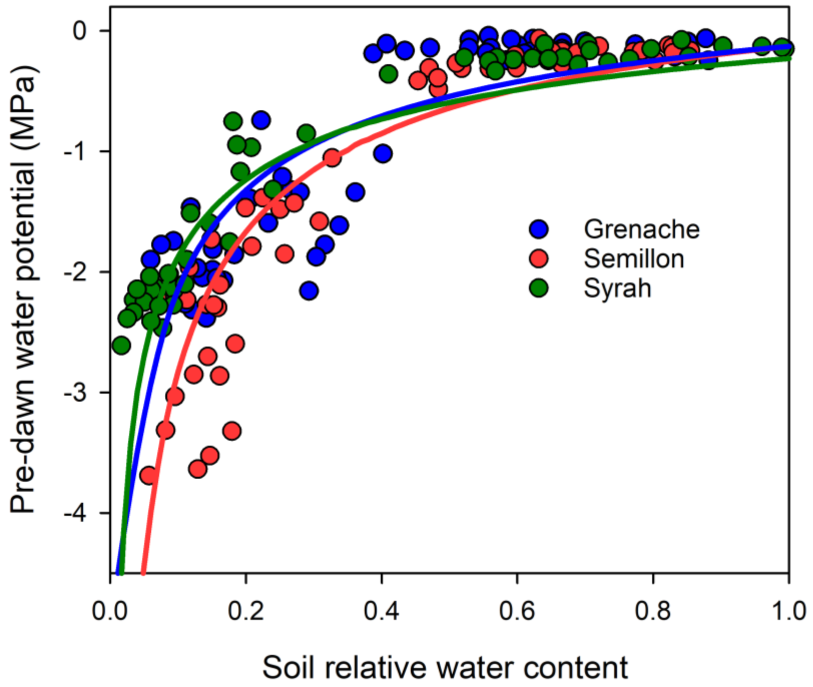

Fig. S3 Variability of diurnal whole plant transpiration values (*E*; mol.m-2.s-1) normalized to leaf area in three grapevine cultivars (Semillon, Grenache and Syrah) in a drought experiment in greenhouse. A) histogram of frequencies B) boxplot.

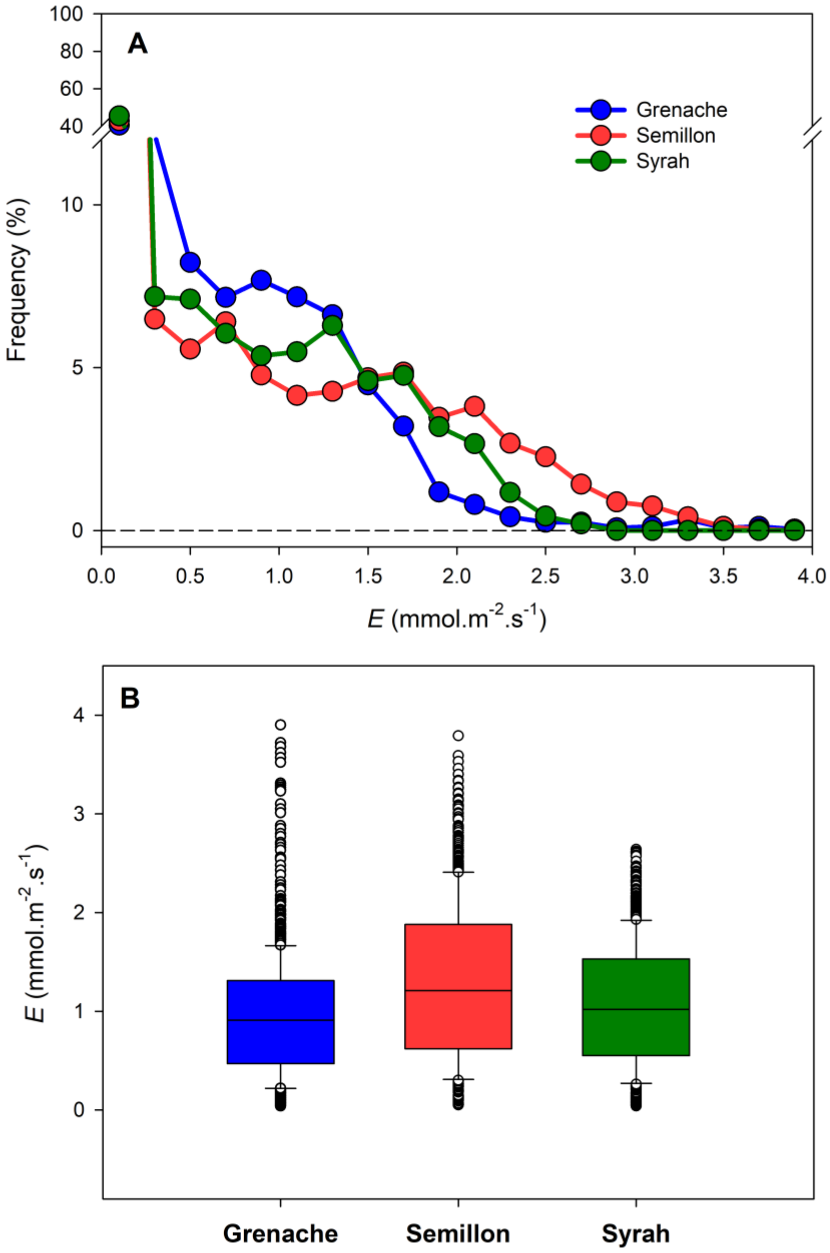

Fig. S4. Minimum leaf conductance measured by the mass loss of detached leaves method in well-watered plants of Grenache, Semillon and Grenache.

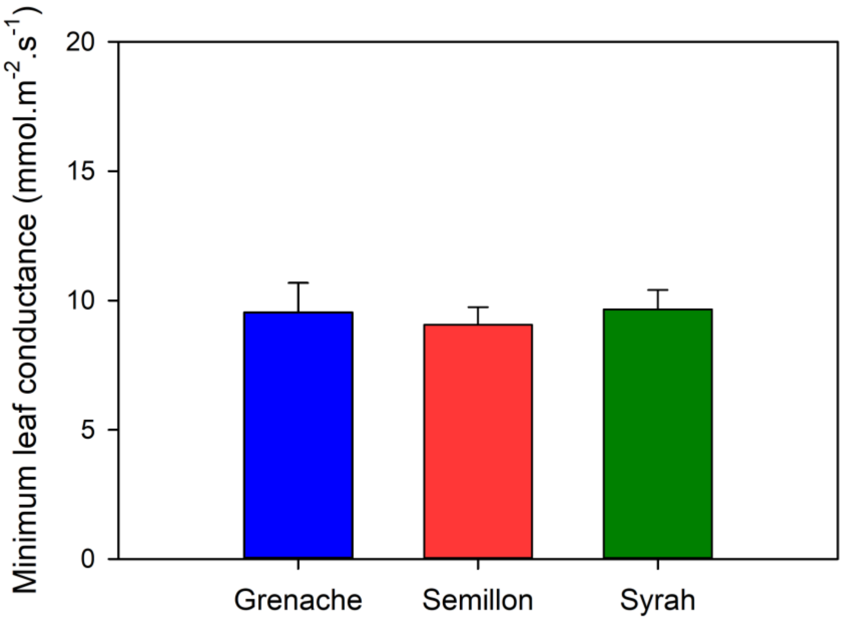

Fig. S5. Pearson correlations between the measured hydraulic traits for the three cultivars.

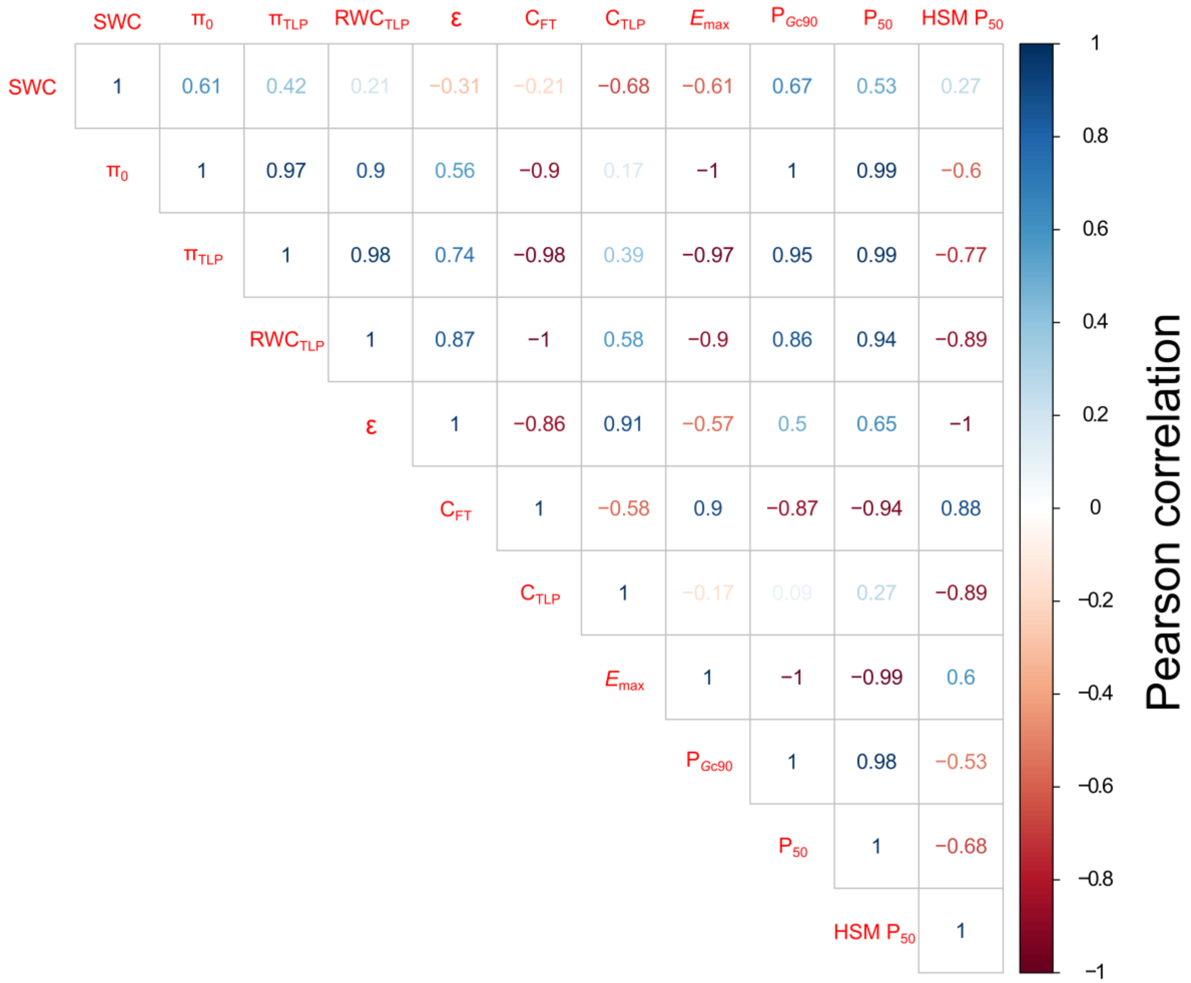

Fig. S6 Simulated declining of maximum water use (E_{plant}; dotted lines) and increasing loss of hydraulic conductivity (PLC%; solid lines) over time (days) under changing environmental conditions (day/time fluctuations) during the progression of drought in three grapevine cultivars (Grenache, Semillon and Syrah) using the 'Sur-Eau' model (Martin-StPaul *et al*., 2017).

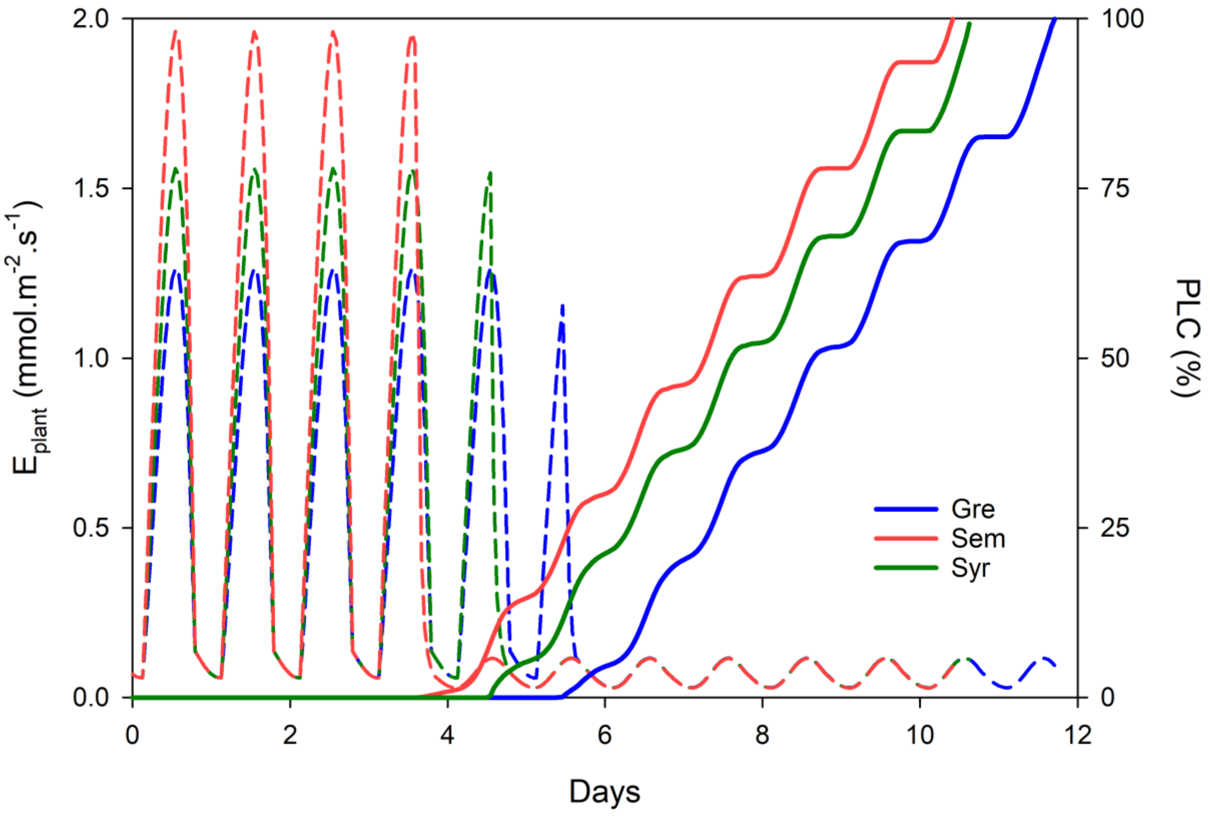

Supplement: eraa186_suppl_Supplementary_Figures [file eraa186_suppl_supplementary_figures.pdf]
